# Neuromechanical adaptations of foot function when hopping on a damped surface

**DOI:** 10.1152/japplphysiol.00012.2022

**Published:** 2022-10-13

**Authors:** Jonathon V. Birch, Dominic J. Farris, Ryan Riddick, Andrew G. Cresswell, Sharon J. Dixon, Luke A. Kelly

**Affiliations:** ^1^Sport and Health Sciences, College of Life and Environmental Sciences, University of Exeter, Exeter, United Kingdom; ^2^School of Human Movement and Nutrition Sciences, The University of Queensland, Brisbane, Queensland, Australia

**Keywords:** foot biomechanics, intrinsic foot muscles, longitudinal arch, multisegment foot models, quasi-stiffness

## Abstract

To preserve motion, humans must adopt actuator-like dynamics to replace energy that is dissipated during contact with damped surfaces. Our ankle plantar flexors are credited as the primary source of work generation. Our feet and their intrinsic foot muscles also appear to be an important source of generative work, but their contributions to restoring energy to the body remain unclear. Here, we test the hypothesis that our feet help to replace work dissipated by a damped surface through controlled activation of the intrinsic foot muscles. We used custom-built platforms to provide both elastic and damped surfaces and asked participants to perform a bilateral hopping protocol on each. We recorded foot motion and ground reaction forces, alongside muscle activation, using intramuscular electromyography from flexor digitorum brevis, abductor hallucis, soleus, and tibialis anterior. Hopping in the Damped condition resulted in significantly greater positive work and contact-phase muscle activation compared with the Elastic condition. The foot contributed 25% of the positive work performed about the ankle, highlighting the importance of the foot when humans adapt to different surfaces.

**NEW & NOTEWORTHY** Adaptable foot mechanics play an important role in how we adjust to elastic surfaces. However, natural substrates are rarely perfectly elastic and dissipate energy. Here, we highlight the important role of the foot and intrinsic foot muscles in contributing to replacing dissipated work on damped surfaces and uncover an important energy-saving mechanism that may be exploited by the designers of footwear and other wearable devices.

## Graphical abstract

**Figure:**
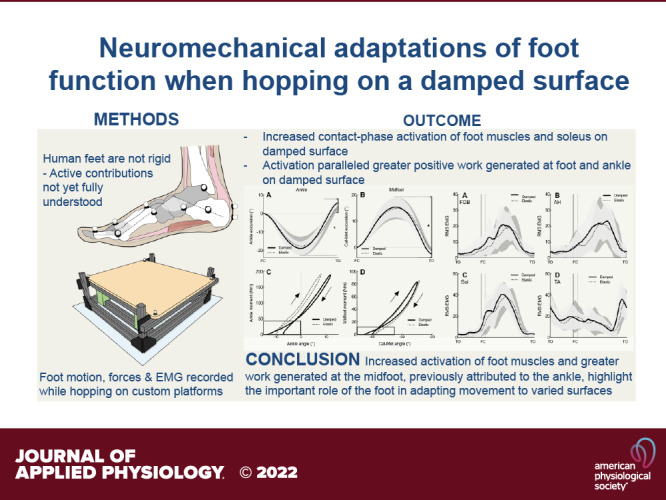

## INTRODUCTION

In bouncing gaits, humans store and return elastic energy in their lower limbs to reduce the muscular work that they must perform to drive their body’s center of mass (COM) forward and upward (1–4). An elastic surface, such as a sprung floor, will also store and return energy. Humans harness this energy by modifying their combined limb stiffness and geometry at contact, reducing the requirement for muscles to perform work to sustain running at a given speed or hopping to a given height (5–9). However, natural substrates are rarely perfectly elastic, meaning that energy is always dissipated to some degree during foot-surface contact (10, 11).

To maintain steady-state motions when interacting with such substrates, humans must replace energy that is dissipated with positive muscle work. Based on human hopping studies on a damped surface, it appears that the ankle plantar flexors are the primary source of this additional mechanical work (10, 11), accounting for twice the combined work that is performed at the knee and hip. However, this research used passive rigid-foot models that ignore the dynamic structures of the foot and can lead to considerable inaccuracies in measures of work and quasi-stiffness about the ankle joint (12, 13). We have previously highlighted the significance of considering the foot as an active, multiarticular part of the lower limb when humans adapt to different surfaces (14). It is therefore important to understand the contribution of the human foot in tuning lower limb mechanics when interacting with a damped surface.

Human feet appear to be an important source of dissipative and generative mechanical work (15–17). During locomotion tasks that require net-positive work to be performed on our COM, foot muscles are actively modulated by the central nervous system to contribute as much as 16% of this work (17, 18). Less is known about how foot function is controlled when moving across surfaces that remove energy from the body. However, we uncovered in prior work that running in cushioned shoes with a viscoelastic midsole leads to an increase in intrinsic foot muscle activation, compared with barefoot running (19). When considered with our recent findings that footwork and muscle activation decrease when hopping on an elastic surface (14), we propose that feet may also play an important role in how humans adjust to operating on surfaces that dissipate energy.

Therefore, the aim of this study was to test the hypothesis that feet perform work to replace energy that is dissipated through collisions with a damped surface. Participants performed a bilateral hopping protocol on a platform that allowed us to alter the damping properties of the surface. When damping of the platform was increased, we expected participants to compensate by increasing the amount of positive work performed within the foot by increasing the activation of the intrinsic foot muscles. We recorded the motion, forces, and muscle activation of the right lower limb and foot to test this hypothesis.

## METHODS

### Participants

Fourteen healthy participants (5 females, 9 males; age, 27 ± 4 yr; height, 170 ± 8 cm; mass, 73 ± 15 kg) volunteered to participate in this study. All participants provided written, informed consent and were free from lower-limb injury in the sixth month before data collection. The experimental procedures were approved by the local ethics review board at The University of Queensland (IRB:2020/HE000456) and were performed in accordance with the Declaration of Helsinki.

### Experimental Protocol

Participants completed a bilateral hopping protocol on elastic and damped surfaces. To manage any learning effect; before data collection, participants were instructed to familiarize themselves with each surface condition. Trials on each surface condition during the familiarization and data collection periods were performed in a counter-balanced order. A digital metronome was used to control hopping frequency at 2.2 Hz, which best represented a preferred frequency for participants (20). Collection for each trial began once it was deemed by the researcher that participants were successfully timing their hops to the beat of the metronome. Participants hopped in place for 30 s and were unshod for all conditions.

### Platform Characteristics

To produce the Elastic and Damped hopping conditions, a modifiable surface was used to alter the mechanical properties of specially fabricated platforms. Three-dimensional ground reaction forces were measured for the right leg using an AMTI force plate (OR6-7; AMTI, Massachusetts). Each platform had identical mechanical properties, only differing in placement within the capture volume; one mounted atop the force plate and one adjacent and level to the first, on the laboratory floor. Each platform was secured in place such that any force applied to the one platform would not interfere with the force readings from the adjacent platform.

**Figure 1.**
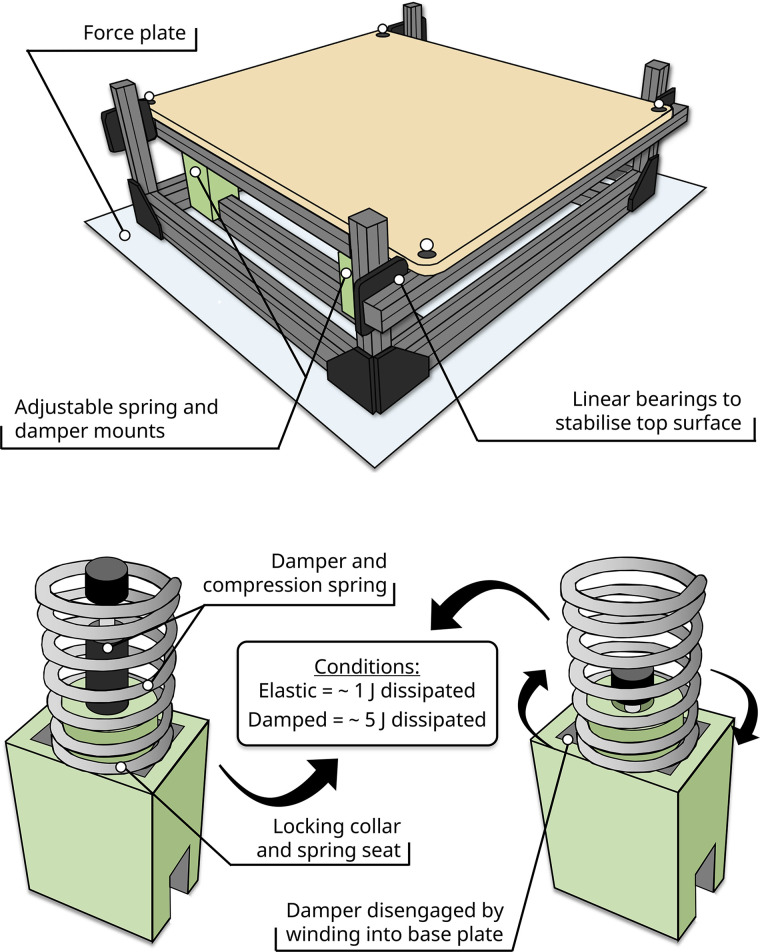
Platform mounting configurations. The right platform was placed atop a force plate for both conditions. Surface damping was altered by engaging and disengaging the shock absorbers located in parallel with the compression springs. The lower panel shows the platform in its damped setting with the compression springs and shock absorbers engaged. For the Elastic condition, the damper was disengaged by winding the locking collar counter-clockwise and turning the damper clockwise into threaded base plate. The locking collar was retained in both conditions to seat the compression spring in place.

Each platform was fabricated using extruded aluminum to produce an upper and lower frame with four linear sliding bearings mounted in each corner to stabilize the upper surface. Fixed to the lower frame was a spring (ASI Springs, Vic., Australia) and damper (MC-75, Ace Controls Inc., Langenfeld, Germany) mounting system that allowed the characteristics of the upper surface to be adjusted between conditions, with the “damped” configuration dissipating ∼5 J of energy per hop and the Elastic condition ∼1 J per hop. The acrylonitrile-butadiene-styrene 3-D-printed housing allowed for the parallel arrangement of both spring and damper in the damped condition and disengagement of the damper for the Elastic condition (Fig. 1). To quantify the characteristics of the Elastic and Damped configurations, the displacement of the top surface was tracked using a motion capture system (see *Kinematic and kinetic measurements*). The energy dissipated in each platform configuration was determined based on force-displacement curves. The area under the curve from maximum platform compression to take off (TO) was subtracted from the area under the curve from foot contact (FC) to maximum compression.

### Data Acquisition

#### Kinematic and kinetic measurements.

The position of retroreflective markers located on the platform and over anatomical landmarks on the right shank and foot of participants was tracked at 200 Hz using an 11-camera optical motion capture system (Qualisys AB, Gothenburg, Sweden). Foot markers were positioned in accordance with the Istituto Ortopedico Rizzoli (IOR) foot model [Leardini et al. (21)]. To minimize unwanted artifacts, markers were attached using adhesive spray and double-sided tape, and where possible further secured with a cohesive bandage. Motion data were synchronously recorded with ground reaction forces and muscle activation data via the Qualisys A/D board.

#### Muscle activation measurements.

Bipolar fine-wire intramuscular electrodes (0.051 mm, stainless steel, Teflon coated; Chalgren Enterprises, California) were inserted under sterile conditions and in accordance with previously described B-mode ultrasound-guided insertion techniques (22) into the muscle tissue of abductor hallucis (AH) and flexor digitorum brevis (FDB) in the right foot of each participant. Ag/AgCl surface electrodes (Covidien LLC., Massachusetts) were placed in accordance with SENIAM guidelines over soleus (Sol) and tibialis anterior (TA) to record surface electromyography (EMG) from the right leg of each participant. All EMG channels were sampled at 4 kHz, amplified 1,000 times, hardware filtered with a bandwidth of 20–2,000 Hz, and grounded with a reference electrode placed over the tibial tuberosity. Preamplifiers and cabling were secured using a cohesive bandage to prevent movement artifacts in the EMG signals.

### Data Analysis

#### Kinematics and kinetics.

**Figure 2.**
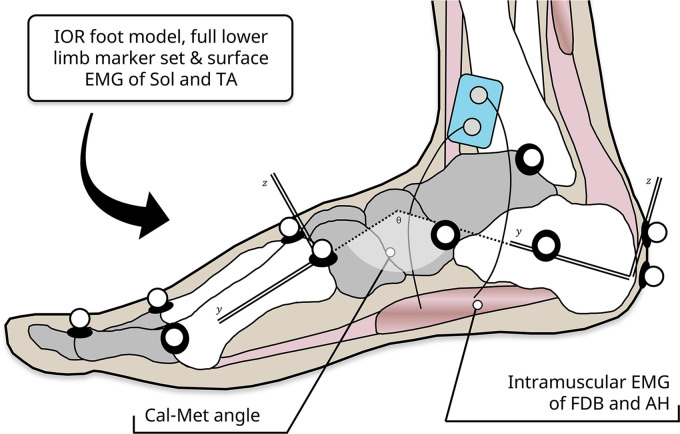
Medial view of experimental set-up on right foot. Medial view of right foot showing IOR foot marker locations and definition of Cal-Met angle. Cal-Met, metatarsal segment with respect to the calcaneus; IOR, Istituto Ortopedico Rizzoli.

Marker position data were digitally filtered using a 10-Hz recursive second-order low-pass Butterworth filter and used to define and scale a rigid body model of the shank, calcaneus, midfoot, metatarsal, and hallux segments for each participant. From this, six degree of freedom representations of the midfoot and ankle could be determined. Sagittal plane motion recorded using this approach shows good agreement with segment positions recorded using biplanar video radiography (23). A joint angle was defined at the midfoot, as the orientation of the metatarsal segment with respect to the calcaneus (Cal-Met angle), with a positive change in the angle representing dorsiflexion of the metatarsals relative to the calcaneus, i.e., compression of the longitudinal arch (Fig. 2). This functional joint represents the combined angular rotation of all the small joints in the midfoot region of the foot (19, 24). The ankle angle was computed as the orientation of the calcaneus relative to the shank as per recent recommendations (12, 13). Joint moments were calculated in Visual3D using an inverse dynamics solution. Mechanical work was calculated as the area under the moment-angle curve for each joint. Ground reaction forces were digitally filtered with a 35 Hz recursive second-order low-pass Butterworth filter, and using a vertical threshold of 50 N, to determine the start and end of each hop cycle. The excursion of the COM during each hop was calculated by twice integrating the net force of each participant with respect to time during each hop (25). Leg stiffness was calculated as the ratio of the peak vertical ground reaction force to the change in length of the leg spring during contact. The resting length of the leg spring was defined as the straight line distance between markers located on the pelvis and metatarsal heads at the instance of each hop contact. Duty factor was calculated as the ratio of contact time to hop duration. Data were then exported to MATLAB (The MathWorks Inc., MA) for subsequent analyses.

#### Muscle activation.

Following direct current offset removal, all EMG signals were digitally filtered: intramuscular channels high-pass at 35 Hz; surface channels, band-pass between 35 and 400 Hz. EMG envelopes of the resultant signals were generated by calculating the root mean square (RMS) amplitude over a moving window of 50 ms and normalized to the maximum amplitude recorded for the respective muscle recorded during a control condition on an infinitely stiff surface. The normalized RMS envelopes were then integrated with respect to time from foot from take off (TO) to foot contact (FC) and from foot contact to take off to yield an integrated EMG value during flight (iEMG_flight_) and contact (iEMG_contact_).

### Statistics

Statistical analyses were performed in GraphPad Prism 9 software (GraphPad Software Inc., CA). Data were checked for normal distribution and paired-sample *t* tests were used to test the influence of surface damping on measures of foot and ankle work and muscle activations. An α level of *P* ≤ 0.05 was used to determine statistical significance. Results are presented as means ± standard deviation (SD) unless otherwise stated.

## RESULTS

### Global Hopping Parameters

Participants preserved the vertical excursion of their COM on both surface conditions (Table 1). In the Damped condition, participants spent significantly longer in contact with the platform surface than they did in the Elastic condition (*P* = 0.02). Since the participants matched the metronome beat on both surfaces, the increased contact time on the damped surface resulted in an increase in duty factor (*P* = 0.01; Table 1).

**Table 1.** Global hopping parameters

	Surface Configuration	
	Elastic	Damped
Actual frequency, Hz	2.2 ± 0.1	2.2 ± 0.4
Ground contact time, ms	304 ± 44	316 ± 46*
Duty factor	0.67 ± 0.1	0.70 ± 0.1*
Net COM excursion, mm	17 ± 1	17 ± 1

Values are group means ± SD; *n* = 13 subjects.

* Significant difference between surfaces *P* < 0.05. COM, center of mass.

### Ankle Mechanics

**Figure 3.**
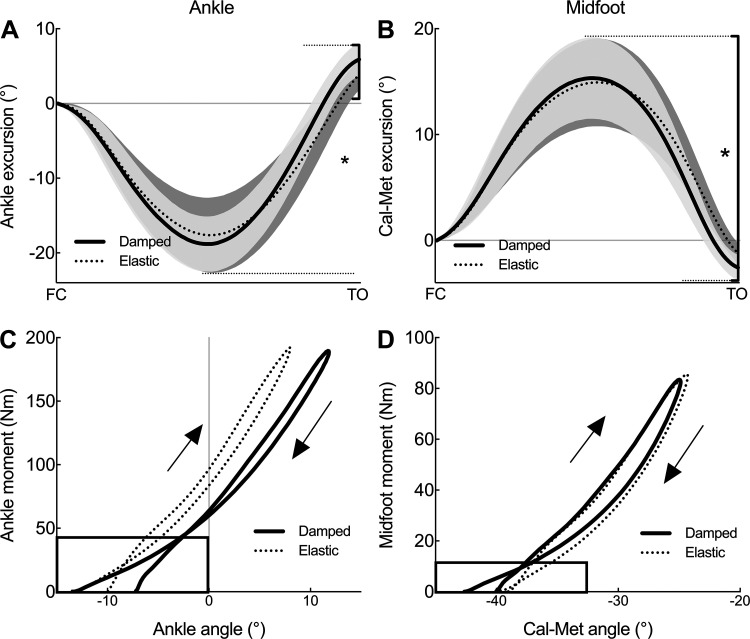
Group means ± SD data (*n* = 13) for the change in angle at the ankle (*A*) and midfoot (Cal-Met; *B*) from foot contact (FC) to toe-off (TO) for both the Damped (solid line) and Elastic (dotted line) conditions. Black bars and asterisk indicate significantly different net ankle plantar flexion and midfoot recoil, respectively. Group data for changes in ankle and midfoot angle vs. joint moment for the ankle (*C*) and midfoot (*D*) for the same Damped and Elastic conditions. Boxes highlight the significantly greater positive work performed in the Damped vs. Elastic condition performed immediately prior to take off. Positive change in angle indicates ankle dorsiflexion and midfoot compression, respectively.

Compared with the Elastic condition, participants plantar flexed and dorsiflexed their ankles more during platform contact with the damped surface condition. Approximately 15% increase in plantar flexion excursion resulted in a 51% greater net plantar flexion excursion at the ankle compared with the elastic surface (*P* = 0.03; Table 2, Fig. 3*A*). This resulted in participants generating more positive work about their ankles in the damped surface condition (*P* = 0.02), which is evident in the ankle angle versus ankle moment plot, immediately before take off (Fig. 3*C*).

**Table 2.** Means ± SD, kinematics, and kinetics per hop for the elastic and damped surface

	Surface Configuration	
	Elastic	Damped
Platform		
Energy dissipated, J/kg	0.02 ± 0.01	0.07 ± 0.02
Net displacement, mm	−1.0 ± 1.0	−5.0 ± 1.0
Stiffness, kN/m	108 ± 6.7	117 ± 9.1
Ankle		
Angle at contact, degrees	−9.0 ± 3.9	−8.2 ± 4.2
Dorsiflexion excursion, degrees	17.6 ± 5.0	19.0 ± 3.6
Plantar flexion excursion, degrees	21.5 ± 4.4	24.8 ± 4.3
Net excursion, degrees	3.9 ± 2.1	5.90 ± 2.2*
Positive work, J/kg	0.36 ± 0.1	0.42 ± 0.1*
Midfoot		
Angle at contact, degrees	−39.1 ± 6.0	−39.4 ± 5.2
Compression excursion, degrees	14.9 ± 4.1	15.4 ± 3.8
Recoil excursion, degrees	16.2 ± 4.5	18.0 ± 4.3
Net excursion, degrees	1.2 ± 1.0	2.6 ± 1.2*
Positive work, J/kg	0.11 ± 0.1	0.13 ± 0.1*

*n* = 13 subjects; mass for normalization = 73 ± 15 kg.

* Significant difference between surfaces *P* < 0.05.

### Foot Mechanics

In a similar manner to the ankle, participants compressed and recoiled their midfoot to a greater degree during contact with the damped surface. Notably, this resulted in net recoil of the midfoot being 115% greater for the Damped condition (*P* = 0.03; Table 2, Fig. 3*B*), allowing participants to generate significantly more positive work about their midfoot for the Damped condition (*P* = 0.02; Table 2). This difference can be seen in the midfoot angle versus midfoot moment plot toward the end of the contact phase of the hop (Fig. 3*D*).

### Muscle Activation

**Figure 4.**
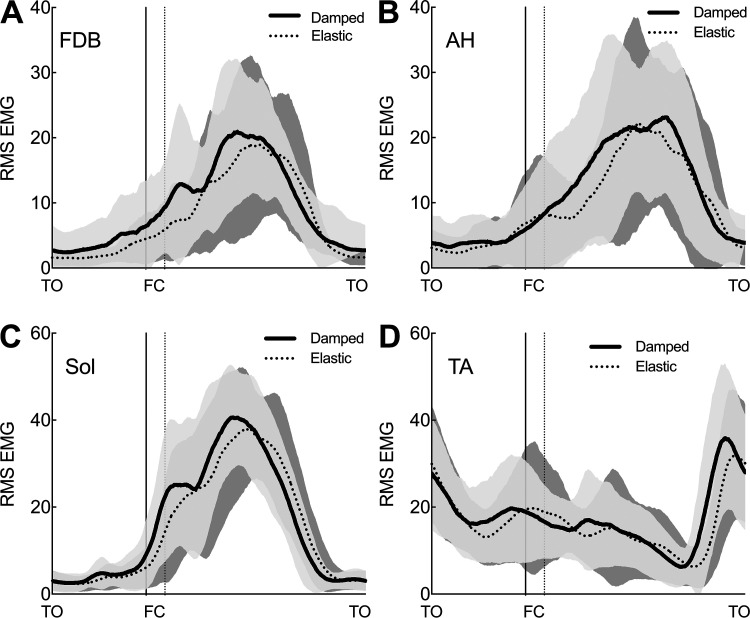
Group mean ensembles ± SD (shaded area) for normalized root mean square (RMS) EMG signal amplitude for flexor digitorum brevis (FDB; *A*), abductor hallucis (AH; *B*), soleus (Sol; *C*), and tibialis anterior (TA; *D*) for the damped (solid line) and elastic (dotted line) surface conditions. Ensembles are presented for a single hop cycle, i.e., from toe-off (TO) to toe-off. Contact with the surface (FC) is indicated by vertical dashed lines to highlight changes in duty factor between conditions (Elastic = dotted line, Damped = solid line). For each muscle, data are normalized for each subject to the peak amplitude recorded on a locked surface.

Sol, AH, and FDB muscles displayed similar overall patterns of activity (increase in integrated EMG) for the Damped as compared with Elastic condition. Increasing activity before contact was followed by a burst of activity during contact (Fig. 4). Tibialis anterior also displayed a burst of activity on the Damped condition immediately before TO. Integrated EMG during contact revealed greater activation of FDB (*P* = 0.02), AH (*P* = 0.02), SOL (*P* = 0.02), and TA (*P* = 0.02) on the Damped compared with the Elastic condition (Table 3).

**Table 3.** Mean ± SD integrated EMG of normalized RMS signal amplitudes

	Surface Configuration	
	Elastic	Damped
Flexor digitorum brevis		
EMG_contact_ (iEMG)	13.42 ± 3.78	14.29 ± 3.50*
EMG_flight_ (iEMG)	0.55 ± 0.36	0.71 ± 0.39
Abductor hallucis		
EMG_contact_ (iEMG)	13.47 ± 3.78	14.29 ± 3.44*
EMG_flight_ (iEMG)	0.56 ± 0.36	0.73 ± 0.40
Soleus		
EMG_contact_ (iEMG)	13.55 ± 3.82	14.43 ± 3.52*
EMG_flight_ (iEMG)	0.55 ± 0.35	0.72 ± 0.40
Tibialis anterior		
EMG_contact_ (iEMG)	13.43 ± 3.79	14.29 ± 3.46*
EMG_flight_ (iEMG)	0.60 ± 0.37	0.78 ± 0.43

*n* = 12 subjects.

*Significant difference between surfaces *P* < 0.05. EMG, electromyography; iEMG, integrated EMG.

## DISCUSSION

During terrestrial locomotion, energy is dissipated during contact with the ground. To maintain constant COM dynamics, we must replace this dissipated energy by activating our muscles to perform positive work. The contribution of our ankle plantar flexor muscles to this function is well described, but the contribution from our feet is unknown, despite being the interface between our body and the substrates we move across. Here, we highlight the structures contained within the foot, specifically, the intrinsic muscles, which contribute a substantial proportion of the additional positive mechanical work required to help offset the energy removed from a damped surface.

Our participants preserved the excursion of their COM when adjusting to the damped surface by increasing the activation of their muscles to generate additional positive work. This was not as necessary in the Elastic condition since the platform surface had recoiled closer to its resting height at take off and assumed a greater portion of the work required to hop to a given height and frequency. In prior work, we observed hopping humans alter their landing geometry when transitioning from a stiff to an elastic surface to harness stored energy, reducing the requirement for active contributions from muscle (14). However, in the current study, we detected no change in landing geometry between surfaces, despite the Elastic setting storing and returning more energy. Since the platform surface in both Damped and Elastic conditions displaced similarly, participants were not able to alter their landing geometry while preserving their COM. That our participants maintained their COM trajectory in agreement with findings reported previously for elastic and damped surfaces (6, 8, 10, 11).

To replace the energy dissipated in the Damped condition, participants altered their foot and ankle mechanics and spent more time in contact with the surface of the platforms. The strategy used by hoppers involved plantar flexing their ankles and recoiling their arch more during the upward phase of the hop cycle. As hypothesized, this resulted in significantly greater positive work being generated by the foot and ankle with respect to the Elastic condition, which paralleled our observation of increased contact-phase intrinsic foot muscle and soleus activation. This work-generating strategy has been observed previously at the ankle (11) and matches trends reported elsewhere in the lower limb where hoppers choose to activate their muscles to extend their joints more in the upward phase of the hop cycle than they are flexed (10, 11). Although the ankle contributed much of the increase in positive work performed in the Damped condition, the intrinsic foot muscles were able to contribute significant portion of this work (25% of that performed at the ankle). That the foot can contribute to replace energy dissipated by a damped surface is a novel finding and when considered with the other recent work from our group (13, 14, 17, 18, 24, 26), further highlights the versatility of our feet in the control of movement.

Qualitatively, the increase in muscle activation that we detected in the Damped condition occurred earlier in the hop cycle than did the burst of positive work at the foot and ankle, which was evident immediately before take off. This pattern, coupled with the significant in-series compliance of the intrinsic foot muscles (16) and ankle plantar flexors (27), points toward participants modulating their muscle-tendon interaction and utilizing stored elastic energy to fulfill/assist with the positive work requirements of the Damped surface. This is consistent with in vivo (28, 29) and simulation (30) data of distal muscle-tendon unit contractile mechanics during positive work generation, whereby the transfer of greater active fascicle shortening to external work is delayed via elastic recoil. Noteworthy is the magnitude of work performed by the foot, with the midfoot accounting for one-third of that performed at the ankle. Owing to simplified modeling techniques, prior studies have attributed this work in its entirety to the ankle joint. As a consequence, its role in adapting leg mechanics to damped surfaces may have been overestimated (12–14). These findings underline the importance of adopting multisegment modeling approaches when considering surface adaptations.

In a previous paper, we observed that running in cushioned shoes with a viscoelastic midsole induced an increase in intrinsic foot muscle activation compared with barefoot running. In the absence of foot kinetics, we hypothesized that this finding was an effort by participants to stiffen the longitudinal arch of their feet to maintain an invariant system stiffness (19). However, in subsequent work, we found that participants actually reduce intrinsic foot muscle activation when hopping on an elastic surface, utilizing stored energy from the surface to contribute to COM dynamics (14). Given that cushioned shoes with viscoelastic midsoles are known to dissipate as much as 35% of the energy stored from midsole compression (31), the results of the current study suggest that the increase in foot muscle activity may have been an effort to replace energy dissipated during midsole compression. The data presented here, highlight that the response from our central nervous system to control foot and ankle mechanics is highly dependent on the properties of the material beneath our feet. Our findings also provide a plausible explanation as to why shoes with lightweight, thick, and highly resilient (elastic) midsoles may provide an energetic advantage during running (31).

While not a gait readily used by humans, we chose to study hopping for a number of reasons. It shares many mechanical similarities with running, relies primarily on the ankle as its source of mechanical power, and could be closely controlled across participants by imposing a frequency constraint. Despite this, future work studying human foot function when running across a damped surface is encouraged and may help to explain adaptations seen in shoes with varied mechanical properties. Our platforms allowed us to carefully parse the difference between damping and stiffness on foot function and intrinsic foot muscle activation. To detect only changes to surface damping, the stiffness of both conditions was closely matched (see Table 2). This limited their capacity to remove energy compared with those previously described (10, 11, 32) and as a result, the magnitude of work generation that we observed at the ankle was far less than in prior work. Our platforms did, however, remove a comparable amount of energy with a cushioned running shoe and as a consequence, our findings may provide insight as to how we adjust to a real-world scenario. We cannot discount the role of extrinsic foot muscles, such as flexor hallucis longus, in generating some of this work. However, when considering the findings of studies that have blocked the ability of the intrinsic foot muscles to contract (18, 24), the extra positive work performed about the midfoot on the damped surface is well within the realms of what the intrinsic muscles are capable of generating.

### Conclusions

In summary, we have presented evidence that the human foot contributes substantial positive work to replace that dissipated by a damped surface via active contributions from the intrinsic foot muscles. These findings support recent work from our group highlighting the foot as an important source of generative work when required and emphasizing that the energetic function of the foot is versatile and tuned based on our interaction with our environment. Our results also offer insight into an energy-saving mechanism that may be exploited by designers of footwear and other wearable devices. Appreciating the important contribution of our feet should be a fundamental consideration in understanding how humans control movement across varied surface requirements.

## GRANTS

This work was supported by the Australian Research Council Discovery Early Career Research Award DE200100585 (to L. A. Kelly), QUEX Institute Scholarship (to J. V. Birch), and Australian Research Council Linkage Grant LP160101316 (to L. A. Kelly, A. G. Cresswell, and D. J. Farris).

## DISCLOSURES

No conflicts of interest, financial or otherwise, are declared by the authors.

## AUTHOR CONTRIBUTIONS

J.V.B., D.J.F., R.R., A.G.C., and L.A.K. conceived and designed research; J.V.B. performed experiments; J.V.B. analyzed data; J.V.B., D.J.F., and L.A.K. interpreted results of experiments; J.V.B. prepared figures; J.V.B. drafted manuscript; J.V.B., D.J.F., R.R., A.G.C., S.J.D., and L.A.K. edited and revised manuscript; J.V.B., D.J.F., and L.A.K. approved final version of manuscript.
